# Associations of Social Cohesion and Socioeconomic Status with Health Behaviours among Middle-Aged and Older Chinese People

**DOI:** 10.3390/ijerph18094894

**Published:** 2021-05-04

**Authors:** Zeyun Feng, Jane M. Cramm, Anna P. Nieboer

**Affiliations:** 1 Erasmus School of Health Policy & Management, Erasmus University Rotterdam, 3000 DR Rotterdam, The Netherlands; Cramm@eshpm.eur.nl (J.M.C.); nieboer@eshpm.eur.nl (A.P.N.); 2 Shanghai Health Development Research Center (Shanghai Medical Information Center), Shanghai 200031, China

**Keywords:** social cohesion, socioeconomic status, physical activity, healthy diet, smoking, social participation, health behaviour

## Abstract

*Background*: An understanding of factors associated with health behaviours is critical for the design of appropriate health promotion programmes. Important influences of social cohesion, education, and income on people’s health behaviours have been recognised in Western countries. However, little is known about these influences in the older Chinese population. *Objective*: To investigate associations of social cohesion and socioeconomic status (SES) with health behaviours among middle-aged and older adults in China. *Methods*: We used data from the World Health Organization’s Study on Global AGEing and Adult Health. Logistic regression and multivariate linear regression were performed. *Results*: Participants who reported greater social cohesion were more likely to have adequate vegetable and fruit (VF) consumption, be socially active, and less likely to smoke daily, but were not physically more active; participants with lower education levels were less likely to have adequate VF consumption and be socially active, and more likely to smoke daily; higher incomes were associated with decreased odds of daily smoking, increased odds of adequate VF consumption, increased likelihood to be socially active, but also less likelihood to have sufficient physical activity (PA). Associations of social cohesion and SES with health behaviours (smoking, PA, and VF consumption) differed between men and women. *Discussion*: Our findings are an essential step toward a fuller understanding of the roles of social cohesion and SES in protecting healthy behaviours among older adults.

## 1. Introduction

China, the country with the largest ageing population on Earth [1], is facing multiple health challenges [2]. Health deteriorates as people age, with increasing disease risk. Healthy behaviours are expected to slow health deterioration by preventing people from becoming ill, as well as by preventing the worsening of chronic illness [3]. Given the importance of leading a healthy lifestyle among older people in China, investigation of the factors associated with health behaviours is critical, and can be particularly useful for the prioritization of limited resources and targeting of public health interventions in the country.

Socioeconomic status (SES), conceptualised as education and income, has been found to be associated with health behaviours [4,5]. Among Chinese adults, for example, less-educated people report lower levels of vegetable and fruit (VF) consumption [6] and higher levels of smoking [7]. People with lower incomes also reported inadequate VF consumption [8]. Diverse mechanisms underlie the relationships between SES disparities and unhealthy behaviours [5]. One classic explanation, termed the “healthy lifestyle” mechanism, is that adults with higher educational levels tend to avoid unhealthy behaviours (e.g., smoking) and to engage in healthy behaviours (e.g., exercise) because education enables people to be more aware of the health outcomes of their behaviours and to develop stronger self-control [9]. Another explanation is that wealthier adults are able to afford the expenses of gym membership and other leisure time associated with physical activity (PA) [10]. In China, however, associations among income, PA, and smoking are complex; people in rural China with lower incomes reported higher levels of work-related PA than did those with higher incomes [11]. Another study showed that women with lower incomes reported higher levels of domestic PA compared with the higher-income group [12]. Possible explanations are that poorer rural residents must work for longer periods to earn livings; women with lower incomes are more likely to be stay-at-home housewives and thus participate more in domestic chores. Additionally, the association between income and smoking is not straightforward. How can we explain the fact that more than half of highly educated doctors in some areas of China are smokers, despite their knowledge of the harmful effects of smoking [13]? This phenomenon indicates that education and income alone are not sufficient to explain people’s health behaviours. Factors other than SES disparities must empower people to adopt certain health behaviours; research has suggested that social circumstances [5] and social environmental factors such as social cohesion [14] can greatly influence such behaviours.

Empirical studies have highlighted the significant influence of social cohesion on people’s health behaviours in Western countries [15,16,17,18,19]. For example, higher levels of social cohesion are associated with higher physical activity (PA) levels among older adults [16,17,18]. Social cohesion can promote PA in many ways [19]. More cohesive societies may be more likely to organize local activities, including sports/PA, that provide more opportunities for residents to adopt and maintain healthy behaviours [20,21]. Social cohesion also may reinforce healthy norms [21]; for example, seeing neighbours jog every day might encourage others to participate in such activities when the perceived safety level (an element of social cohesion) is high [17].

This mechanism may also apply to the maintenance of a healthy diet. Collective efficacy, another aspect of social cohesion, is grounded in mutual trust and describes a community’s ability to create change and exercise informal social control (e.g., promote healthy vegetable and fruit (VF) consumption through social norms) [22]. Several scholars have found that greater social cohesion is associated with higher VF intake among adults [23] and adolescents [24], and benefits nutrition among children [25]; little attention has been given to this association in older adults. In a study conducted with 5900 adults living in urban neighbourhoods in five European countries, higher levels of social cohesion were associated positively with fruit, but not vegetable, intake [26].

The relationship between social cohesion and smoking appears to be less straightforward, as studies evaluating it have yielded different conclusions; some researchers found that greater social cohesion was associated with lower levels of smoking [27,28,29], whereas Andrews and colleagues [30] found no such association.

Apart from traditional health behaviours, social participation has also been reported recently to be a crucial health behaviour in later adulthood [31]. Studies conducted in Western countries, such as Great Britain [22,32] and the United States [33], have revealed a clear association between social cohesion and social participation among older adults, although evidence on this subject remains scarce, and whether this association holds among older adults in China remains unknown.

Numerous attempts have been made to conceptualize social cohesion [34,35]. In general, the term refers to trust levels and the absence of social conflict, interrelated societal characteristics [36,37], but an internationally accepted definition remains lacking. For this study, we adopted Chan and colleagues’ [38] (pp. 290) definition: “social cohesion is a state of affairs concerning both the vertical and the horizontal interactions among members of a society, as characterized by a set of attitudes and norms that include trust, a sense of belonging, and the willingness to participate and help, as well as their behavioural manifestations.” Researchers have proposed several indicators for its measurement [39,40], including trust among citizens [17,18,21,39,40,41,42] and perceived safety [39], which are expected to influence health behaviours.

Despite China’s rapid economic growth in recent decades, the income gap (reflected by the Gini coefficient) in the country is ranked even higher than that in the United States [43]. It peaked in 2008 and then began to decline in 2010 [43]. According to the Committee on Social Affairs, Health and Sustainable Development (Council of Europe), a substantial body of evidence has shown that income inequality is a major threat to social cohesion [44]. The drastic economic development that has occurred in the past few decades in China has likely affected social cohesion. Thus, the investigation of social cohesion in China during the period of 2008–2010 is of particular interest.

Research investigating associations between social cohesion, SES, and health behaviours among older people in China is very limited; only one study revealed an association between social cohesion and leisure-time physical activity (LTPA) among older adults in Shanghai [21]. No study to date has explicitly examined associations of social cohesion and SES with multiple health behaviours in a national sample of older Chinese people. Although the importance of SES has been well documented in developed nations [5], less evidence is available for developing countries such as China. To fill this gap, we investigated associations of social cohesion and SES with various health behaviours (smoking, physical activity, VF consumption, and social participation) among middle-aged and older adults in China using a large nationwide database. As previous studies have revealed gender differences in health behaviours such as smoking in China [45], we also conducted a gender-stratified analysis of these associations.

## 2. Methods

### 2.1. Participants and Data

Data from Chinese participants in wave 1 (2008–2010) of the World Health Organization’s (WHO’s) Study on Global AGEing and Adult Health (SAGE) were used for the current study, which is the most recent available data from China. This period is also of interest because income inequality in China peaked in 2008 and only began to decline in 2010 [43]. SAGE is a nationally representative study of individuals aged ≥50 years in six low- and middle-income countries (China, Ghana, India, Mexico, the Russian Federation, and South Africa). In China, the wave 1 survey was conducted in between 2008 and 2010 in 8 provinces/municipalities [46]. A multistage, stratified cluster sampling approach was used to select participants [46]. Approximately half of the face-to-face interviews were computer-assisted (CAPI), and half were assisted by manual data recording [46]. The individual response rate was excellent (93%) [46]. Further details of WHO SAGE sampling have been provided elsewhere [47]. The sample for this study comprised 13,367 participants.

### 2.2. Measures

#### 2.2.1. Independent Variables

##### Social Cohesion Scale

Social cohesion was operationalized by using a mean scale based on respondents’ answers to five questions about trust and safety developed by WHO SAGE as a social cohesion indicator: neighbourly trust, trust in co-workers, trust in strangers, perceived safety while staying alone at home, and perceived safety while walking alone in streets after dark (details shown in Appendix A). The original questionnaire requires respondents to rate the levels of trust/safety on a five-point scale. In our analyses, all answers were inverse-coded for convenience of interpretation. Meaning, for trust items, each answer was based on a five-point scale, ranging from 1, denoting “to a very small extent” (1), to 5, “to a very great extent” (5); for safety items, answers ranged from “not safe at all” (1) to “completely safe” (5). At least three out of five items needed to be answered. Higher scores indicated higher levels of social cohesion.

##### SES

Based on previous research [5,48,49], education and income were used to measure SES in our analyses. Individuals’ educational levels were recorded as lower (completed primary school or less: 0) and higher (completed secondary school or more: 1). Individuals’ incomes were estimated by the WHO SAGE research team. The Bayesian postestimation method was used to estimate raw income based on income indicators such as various dwelling characteristics (e.g., type of floor), a set of household ownership of durable goods (e.g., number of chairs), and access to services (improved water, sanitation, and cooking fuel) [50].

##### Sociodemographic Characteristics

The following sociodemographic variables were controlled in our analyses: age (years), gender (0, male; 1, female), marital status (0, single (never married, separated/divorced, or widowed); 1, married (currently married or cohabiting)), and area of residence (0, urban; 1, rural).

#### 2.2.2. Dependent Variables

##### PA

PA was assessed using a dichotomous variable based on self-reported questionnaire responses. Participants were asked to report their vigorous and moderate PA. Vigorous PA included work activities (e.g., chopping, farm work, and digging with a spade or shovel) and sports, leisure, and recreational activities (e.g., jogging, running, swimming, heavy lifting, fitness, gym attendance, and rapid cycling). Moderate PA included washing clothes by hand, gardening, house cleaning, stretching, dancing, and cycling at regular pace. Participants were asked to recall the level of activities and the time spent on them in a typical week. We used the WHO-recommended thresholds (for individuals aged ≥18 years) to classify PA as sufficient (≥150 min/week moderate or ≥75 min/week vigorous PA: 1) and insufficient (<150 min/week moderate or <75 min/week vigorous PA: 0) [51].

##### VF Consumption

VF consumption was used as an indicator of healthy eating. We followed the WHO guidelines [51] to distinguish adequate (≥2 servings fruit and ≥3 servings vegetables/day: 1) from inadequate (<2 servings fruit and <3 servings vegetables/day: 0) VF consumption.

##### Smoking

Smoking behaviour was assessed by asking whether participants smoked daily. This variable was dichotomised as 0 (not a daily smoker) and 1 (daily smoker).

##### Social Participation Scale

Social participation was measured using a mean scale for the 9-item questionnaire developed for the SAGE (Appendix B), with questions such as “How often in the last 12 months have you attended any public meeting in which there was discussion of local or school affairs?”. Responses ranging from “never” (1) to “daily” (5) denote the frequency of respondents’ involvement in their communities. Total social participation scores were calculated by summing the item scores.

## 3. Statistical Analysis

As descriptive statistics, means, and standard deviations (SDs) of continuous variables (e.g., age) and numbers and percentages of categorical variables (e.g., gender) were calculated. The strength of associations between social cohesion and health behaviours (categorical variables: PA, VF consumption, and smoking) was evaluated by estimating odds ratios (ORs) with 95% confidence intervals (CIs) using a logistic regression model. The association between social cohesion and social participation (a continuous variable) was evaluated by estimating B coefficients and standard errors (SE) using a multivariate linear regression model. Social cohesion and SES variables (income and education) were entered into the models simultaneously while adjusting for key individual background characteristics (age, gender, marital status, and area of residence). To produce gender-specific analyses, stratified analyses were performed, while adjusting for age, marital status, and areas of residence. To assess the severity of multicollinearity, we calculated the variance inflation factors (VIF) among independents variables. The VIF score of all covariates did not exceed the recommended value of 10 [52]; which suggested that there were no multicollinearity problems among independent variables included in our analyses. The significance level was set at *p* < 0.01. All statistical analyses were conducted using IBM SPSS Statistics (version 27, IBM, Armonk, NY, USA).

## 4. Results

Table 1 shows the characteristics of the study participants. Of the 13,367 participants included, the mean age (SD) was 63.2 (9.44) years; 53.1% of participants were female, 83.1% were not single, 50.9% were from rural areas, and 61.7% had lower educational levels. Overall, the prevalence of smoking was 24.5%, but a much higher proportion of smokers was male (48.9% vs. 3.0% female). The prevalence of inadequate VF consumption was 35.0%, and 32.8% of participants reported insufficient PA. The mean social participation scale score was 1.7 (standard deviation, 0.4).

Table 2 presents the results of the multivariate linear regression model and logistic regression models. In the analysis adjusted for age, gender, marital status, and residence, each unit of increase in the social cohesion score was associated with a 30% increase in the likelihood of adequate VF consumption (OR = 1.300; 95% CI, 1.192–1.417; *p* < 0.001); higher social cohesion was associated with lower odds of being a daily smoker (OR = 0.839; 95% CI, 0.754–0.934; *p* < 0.01); also, higher mean score of social cohesion was positively associated with higher levels of social participation (B = 0.074, *p* < 0.001). Regarding education, less-educated respondents were associated with lower odds of having adequate VF consumption (OR = 0.806; 95% CI, 0.730–0.890; *p* < 0.001), lower-educated respondents had a 31% higher likelihood of being daily smokers (OR = 1.314; 95% CI, 1.166–1.480; *p* < 0.001), and were less likely to be socially active (B = −0.052, *p* < 0.001) compared with people with higher levels of education. With respect to income, individuals with higher income were less likely to have sufficient PA (OR = 0.606; 95% CI, 0.552–0.665; *p* < 0.001), less likely to be daily smokers (OR = 0.790; 95% CI, 0.699–0.891; *p* < 0.001), more likely to have adequate VF consumption (OR = 2.650; 95% CI, 2.396–2.932; *p* < 0.001), and tend to be more socially active (B = 0.101, *p* < 0.001) compared with people with lower income.

Analyses controlled for key background characteristics (age, marital status, and area of residence) revealed significant gender differences in the associations of daily smoking and PA with social cohesion (Table A3, Appendix C). Higher levels of social cohesion were associated significantly with decreased odds of being a daily smoker among men (OR = 0.805, *p* < 0.001), but not among women. Such levels were associated significantly with sufficient PA only among men (OR = 1.178, *p* < 0.01). In addition, gender differences were found in the associations of education with adequate VF consumption and daily smoking (Table A3, Appendix C). Lower educational levels were associated significantly with reduced odds of adequate VF consumption among women (OR = 0.723, *p* < 0.001), but not men. Such levels were associated significantly with greater odds of being a daily smoker only among men (OR = 1.320, *p* < 0.001). In addition, higher incomes were associated significantly with reduced odds of being a daily smoker only among men (OR = 0.807, *p* < 0.01)

## 5. Discussion

In general, this study revealed that older Chinese people with greater social cohesion are more likely to have adequate VF consumption and to be socially active, and less likely to be daily smokers, but were not physically more active. Participants with lower education levels were less likely to have adequate VF consumption and to participate in social activities, and were more likely to be daily smokers than those with more education. Higher incomes were associated with a reduced likelihood of being a daily smoker and increased likelihood of having adequate VF consumption and being socially active, but also a reduced likelihood of engaging in sufficient levels of PA. This study serves as a first step in the deepening of our knowledge of the crucial role of social cohesion for health behaviours among older adults in China.

### 5.1. Associations of Social Cohesion with Health Behaviours

Our finding that greater social cohesion decreased the odds of smoking, which is in agreement with previous research [27,28,29], supports the theory that social cohesion strengthens psychological resources (e.g., self-esteem and optimism) and helps to reduce smoking risk factors, such as distress [29]. Similarly, our finding that older people with greater social cohesion are more likely to be socially active is in accordance with findings from Western countries, such as the United States [33]. No comparable data for older adults in China were available. In highly collectivistic societies, people tend to limit their social activities, including only people in their inner circles; they tend to be comfortable participating in social activities with others only when they feel that they can trust them [53]. Our finding implies that the enhancement of older people’s perceived safety and trust (vital elements of social cohesion) boosts their social participation. The lack of association between social cohesion and PA in this study is consistent with Legh-Jones and Moore’s finding [54] that perceived generalized trust was not associated with PA among adults. However, other researchers have reported a positive association with LTPA [18,21,55]. This inconsistency may reflect the use of different PA measures among studies [19]. To be specific, we included multiple aspects of PA (e.g., gardening, walking, and household chores), whereas Lindström [55], Gao [21], and Van Dyck’s [18] studies focused on the associations between social cohesion and LTPA specifically. Thus, social cohesion may be more relevant for leisure-time activities (e.g., going shopping, going to the movies, and dining at a restaurant) than for other types of PA (e.g., gardening and household chores). Finally, we observed a positive association between greater social cohesion and sufficient VF consumption among older adults. This finding is in line with the findings of a study conducted in Japan, which revealed that people living in more cohesive neighbourhoods more frequently had sufficient VF intakes [56]. Although empirical studies of VF receipt among older adults in China are lacking, older Chinese adults who cultivate VF are likely to more frequently share their products with neighbours they trust as an indicator of greater social cohesion. Previous findings on this topic are inconsistent. Barnidge and colleagues [57] found no significant association between social cohesion and VF consumption, and a multinational study conducted in Europe [26] revealed an association with fruit, but not vegetable, consumption. This discrepancy may be due to the examination of different study populations using different measures; we included middle-aged and older adults living throughout China, whereas Barnidge et al. [57] focused on older adults (mostly women) in rural settings in the United States and Mackenbach and colleagues [26] examined a general adult population from urban areas in Europe. Furthermore, we followed the WHO guidelines to distinguish adequate and inadequate VF consumption as one variable, Mackenbach and colleagues’ [26] study measured fruit consumption and vegetable consumption separately as two variables. Additionally, as admitted by Barnidge and colleagues [57], their study potentially brought bias regarding the reporting of VF consumption because they used a single item to measure VF consumption. Our finding, however, is consistent with the expected presence of such an association, and expands our understanding of it in general older adult populations.

### 5.2. Associations of SES with Health Behaviours

Our finding that older Chinese adults with higher incomes were more likely to be physically inactive is in accordance with previous findings for Chinese adults [11]. Older adults with higher incomes are more likely to own and use (personal) vehicles [58], which decreases their daily engagement in physical activities such as walking and cycling. In addition, this group may be less likely than those with lower incomes to need to engage in physically demanding work, for example, by hiring workers to do household chores. Although we found that higher incomes decreased the risk of being a daily smoker among older Chinese adults, according to Zhang and colleagues’ [59], national Chinese surveys have revealed no relationship between household income and smoking behaviour (among men). This inconsistency might be explained by an age difference among study samples; the national surveys were conducted with adults aged ≥18 and ≥15 years, respectively [60,61]. Although higher education levels have been associated with higher levels of exercise [62], we observed no such association in our overall sample. Age may also explain this discrepancy, as the previous study was conducted with individuals aged 15–69 [62]. In addition, only 31% of participants in Gang et al.’s [62] study had lower educational levels (0–6 years of school), whereas 61.7% of our participants had completed primary school or less. Relationships between education levels and health behaviours need to be examined further.

### 5.3. Gender Specific Findings

This study revealed some gender differences related to smoking, PA, and VF consumption. Lesser social cohesion and lower educational levels and incomes were associated with daily smoking only among older Chinese men. These findings could be explained by the difference in smoking patterns between men and women [63,64], and the corresponding small number of female smokers in our sample. Various surveys have revealed low prevalence rates for smoking among Chinese women [60]. For example, this rate was 2.4% in the 2010 Global Adult Tobacco Survey [65], likely because smoking is an accepted social norm for men, but not women, in China [66]. Greater social cohesion was associated with sufficient PA only among men in this study. In traditional Chinese culture, women are responsible for housework and are thus more likely than men to engage in domestic forms of PA (e.g., cooking and cleaning) [67]. Thus, social cohesion may have less influence on Chinese women’s PA.

Lower educational levels were associated with inadequate VF consumption only among women in this study. A study conducted in Korea revealed an association between lower educational levels and lower VF intake, and specifically low consumption of yellow/orange vegetables in men and red fruit/vegetables in both men and women [68]. Due to differences in study samples and the measurement of VF consumption, comparison of our findings with those of Hong and colleagues [68] is difficult. Evidence regarding gender differences in the associations of social cohesion and SES with health behaviours in China is lacking. While this study provided a first insight into these gender differences, more studies are needed to gain an in-depth understanding of whether and how the mechanisms underlying older adults’ social cohesion and health behaviours differ according to gender.

### 5.4. Public Policy Implication

The findings of this study provide valuable insight for policy development to promote healthy ageing among older adults in China. For instance, investment in the creation of safe neighbourhoods is expected to benefit older adults’ health behaviours. Vest and Valdez [69] found that people who described their neighbourhoods as unsafe were almost three times more likely to be physically inactive than were people describing their neighbourhoods as extremely safe. Health policies should thus aim to create safe, walkable, and accessible neighbourhoods by increasing urban public space (e.g., community gardens and parks) to encourage older adults’ outdoor (physical and social) activities and social interactions [70]. Furthermore, our findings highlight the importance of considering gender differences when designing health promotion strategies aiming to improve older Chinese adults’ health behaviours.

### 5.5. Study Strengths and Limitations

This study contributes to the literature in several ways. First, China’s unprecedented development has created a unique context for social scientists, as the rapid changes that have occurred have had profound impacts on the country’s population. Specifically, scholars believe that economic growth can influence social cohesion [71]. This study is the first in which data from a large population-based sample were used to investigate the associations of social cohesion and SES with various health behaviours among older Chinese people. Second, we minimized bias by controlling for various potential confounders, such as sociodemographic factors, in our regression models.

Notwithstanding, several limitations of this study warrant mention. First, we could not assess causality or changes in social cohesion, SES, or health behaviours, due to the cross-sectional study design. We encourage researchers to explore longitudinal relationships among these factors when wave 2 SAGE data become publicly available. In addition, bundling of health behaviours should be considered, as a previous study showed that people tend to gain weight when they quit smoking due to the consumption of more food/snacks as rewards for smoking withdrawal [72]. Second, we used VF consumption as an indicator of healthy diet due to limited data availability, although VF consumption alone cannot fully reflect individuals’ dietary patterns. Thus, we urge researchers to collect more detailed dietary information according to the WHO guidelines, to augment our ability to assess these patterns. Third, the lack of global consensus on the definition of social cohesion—a well-known problem in this research field—makes the comparison of research findings difficult [41]. Fourth, we did not examine alcohol consumption in this study because face-to-face interviews have been shown to generate socially desirable answers to questions on this topic, with underreporting of alcohol consumption [73]. Lastly, due to data limitation, the measurement of social cohesion was limited to trust and safety indicators. More research is needed to develop an internationally accepted definition of social cohesion and means of operationalising this concept.

## 6. Conclusions

In this study, greater social cohesion was associated with adequate VF intake, active social participation, and not being a daily smoker among middle-aged and older adults in China. Higher educational levels and incomes were associated with favourable health behaviours, except that higher incomes were associated with insufficient PA. Our findings are an essential step toward a fuller understanding of the roles of social cohesion and SES in protecting healthy behaviours among older adults in China. Policymakers and health professionals designing health promotion strategies should aim to enhance social cohesion among middle-aged and older adults in China, which may vary between Chinese older men and women.

## Figures and Tables

**Table 1:** Characteristics of the study population (*n* = 13,367).

	*n*	%	Mean (SD)
**Sociodemographic characteristics**			
Age (years) Range 50–99	13,367		63.2 (9.4)
Gender (female)	7093	53.1	
Marital status Missing 10 (0.1%)			
Non-single	11,093	83.1	
Areas of residence (rural)	6800	50.9	
**SES and social cohesion variables**			
Educational level Missing 72 (0.5)			
Lower	8202	61.7	
Income quintile Missing 61 (0.5)			
Q1 (lowest)	2665	20.0	
Q2	2646	19.9	
Q3	2688	20.2	
Q4	2724	20.5	
Q5 (highest)	2583	19.4	
Social cohesion scale Missing 429 (3.2)	12,938		3.4 (0.5)
**Health behaviours**			
Daily smoker Missing 443 (3.3)			
Female	209	3.0	
Male	2954	48.9	
Total sample	3163	24.5	
Inadequate VF consumption Missing 1247 (9.3)			
Female	2013	28.4	
Male	2223	39.0	
Total sample	4236	35.0	
Insufficient PA Missing 422 (3.2)			
Female	2284	33.2	
Male	1960	32.3	
Total sample	4244	32.8	
Social participation scale Missing 419 (3.1)			
Female	6879		1.7 (0.4)
Male	6069		1.7 (0.4)
Total	12,948		1.7 (0.4)

SD, standard deviation; SES, socioeconomic status; VF, vegetables and fruit; PA, physical activity. No data on age, gender, residence were missing. Higher Social participation scores indicate greater social participation.

**Table 2:** Relationships between social cohesion and socioeconomic status with four health behaviours.

	Sufficient PA	Adequate VF Consumption	Daily Smoker	Social Participation ^§^	
	OR (95% CI)	OR (95% CI)	OR (95% CI)	B	SE
Independent variables					
Social cohesion	1.058	1.300 **	0.839 *	0.074	0.007 **
	(0.975–1.147)	(1.192–1.417)	(0.754–0.934)		
Education (low)	1.058	0.806 **	1.314 **	−0.052	0.008 **
	(0.963–1.162)	(0.730–0.890)	(1.166–1.480)		
Income	0.606 **	2.650 **	0.790 **	0.101	0.008 **
	(0.552–0.665)	(2.396–2.932)	(0.699–0.891)		
Covariates					
Age	0.960 **	1.000	0.959 **	−0.005	0.000 **
	(0.956–0.964)	(0.995–1.005)	(0.953–0.964)		
Gender (female)	0.937	1.510 **	0.027 **	−0.003	0.007
	(0.867–1.013)	(1.390–1.640)	(0.023–0.032)		
Residence (rural)	0.681 **	0.455 **	1.608 **	0.122	0.008 **
	(0.621–0.745)	(0.415–0.500)	(1.431–1.808)		
Non-Single	1.078	1.330 **	0.787 *	−0.003	0.010
	(0.968–1.200)	(1.188–1.489)	(0.671–0.923)		
Constant	25.762 **	0.889	20.600 **		1.679 **
R2	0.047	0.153	0.445		0.062
	(Nagelkerke)	(Nagelkerke)	(Nagelkerke)		
*n*	12,822	12,005	12,797		12,840

* *p* < 0.01, ** *p* < 0.001. SE, standard error; OR, odds ratio; CI, confidence interval. ^§^, continuous variable. PA, physical activity. Reference groups: male, urban residence, single, higher education. Higher Social participation scores indicate greater social participation.

## Data Availability

The data presented in this study are openly available in WHO Multi-Country Studies Data Archive at: https://apps.who.int/healthinfo/systems/surveydata/index.php/catalog/13 (accessed on 2 May 2021).

## Appendix A

**Table A1:** Social cohesion scale.

**How Much You Trust Different Groups of People…**				
To a very small extent	To a small extent	Neither great nor small extent	To a great extent	To a very great extent
First, think about people in our neighbourhood. Generally speaking, would you say that you can trust them…?				
1	2	3	4	5
Now, think about people whom you work with. Generally speaking, would you say that you can trust them …?				
1	2	3	4	5
How about strangers? Generally speaking, would you say that you can trust them …?				
1	2	3	4	5
**Questions about Safety in the Area Where You Live.**				
Not safe at all	Slightly safe	Moderately safe	Very safe	Completely safe
In general, how safe from crime and violence do you feel when you are alone at home?				
1	2	3	4	5
How safe do you feel when walking down your street alone after dark?				
1	2	3	4	5

## Appendix B

**Table A2:** Social participation scale.

How Often in the Last 12 Months Have You…
1. Attended any public meeting in which there was a discussion of local or school affairs?
2. Met personally with someone you consider to be a community leader?
3. Attended any group, club, society, union or organisational meeting?
4. Worked with other people in your neighbourhood to fix or improve something?
5. Had friends over to your home?
6. Been in the home of someone who lives in a different neighbourhood than you do or had…them in your home?
7. Socialised with co-workers outside of work?
8. Attended religious services (not including weddings and funerals)?
9. Gotten out of the house/your dwelling to attend social meetings, activities, programmes or events or to visit friends or relatives?

## Appendix C

**Table A3:** Associations between social cohesion and socioeconomic status with health behaviours among males and females.

	Sufficient PA		Adequate VF Consumption		Daily Smoker		Social Participation ^§^	
	Males	Females	Males	Females	Males	Females	Males	Females
	OR (95% CI)	OR (95% CI)	OR (95% CI)	OR (95% CI)	OR (95% CI)	OR (95% CI)	B (SE)	B (SE)
Social cohesion	1.178 *	0.976	1.251 **	1.357 **	0.805 **	0.984	0.067	0.080
	(1.044–1.329)	(0.874–1.091)	(1.106–1.416)	(1.202–1.531)	(0.716–0.904)	(0.732–1.323)	(0.011) **	(0.010) **
Low education	0.941	1.184	0.866	0.723 **	1.320 **	1.413	−0.050	−0.055
	(0.826–1.073)	(1.034–1.356)	(0.759–0.989)	(0.620–0.841)	(1.163–1.498)	(0.938–2.129)	(0.012) **	(0.012) **
Income	0.595 **	0.616 **	2.506 **	2.806 **	0.807 *	0.652	0.124	0.081
	(0.519–0.682)	(0.541–0.700)	(2.175–2.887)	(2.430–3.240)	(0.708–0.920)	(0.461–0.923)	(0.012) **	(0.011) **
Age	0.969 **	0.952 **	1.010 *	0.990 *	0.948 **	1.037 **	−0.004	−0.006
	(0.963–0.975)	(0.946–0.958)	(1.003–1.017)	(0.983–0.996)	(0.942–0.954)	(1.020–1.055)	(0.001) **	(0.001) **
Residence (rural)	0.840	0.568 **	0.493 **	0.426 **	1.704 **	1.076	0.152	0.098
	(0.736–0.960)	(0.501–0.644)	(0.432–0.563)	(0.373–0.486)	(1.503–1.933)	(0.778–1.488)	(0.012) **	(0.011) **
Non-Single	1.072	1.051	1.277	1.265 *	0.878	0.951	0.035	−0.023
	(0.894–1.286)	(0.916–1.205)	(1.061–1.537)	(1.092–1.466)	(0.708–0.920)	(0.677–1.338)	(0.017)	(0.012)
Constant	9.387 **	55.798 **	0.512	2.640 *	41.854 **	0.002 **	1.607	1.734
							(0.056) **	(0.051) **

* *p* < 0.01. ** *p* < 0.001. SE, standard error; OR, odds ratio; CI, confidence interval. ^§^ continuous variable. PA, physical activity; higher social participation scores indicate greater social participation.
